# Sleep spindles in primates: Modeling the effects of distinct laminar thalamocortical connectivity in core, matrix, and reticular thalamic circuits

**DOI:** 10.1162/netn_a_00311

**Published:** 2023-06-30

**Authors:** Arash Yazdanbakhsh, Helen Barbas, Basilis Zikopoulos

**Affiliations:** Computational Neuroscience and Vision Lab, Department of Psychological and Brain Sciences, Boston University, Boston, MA, USA; Graduate Program for Neuroscience, Boston University, Boston, MA, USA; Center for Systems Neuroscience, Boston, MA, USA; Neural Systems Laboratory, Program in Human Physiology, Department of Health Sciences, College of Health and Rehabilitation Sciences (Sargent College), Boston University, Boston, MA, USA; Department of Anatomy and Neurobiology, Boston University School of Medicine, Boston University, Boston, MA, USA; Human Systems Neuroscience Laboratory, Program in Human Physiology, Department of Health Sciences, College of Health and Rehabilitation Sciences (Sargent College), Boston University, Boston, MA, USA

**Keywords:** First-order thalamic nuclei, High-order thalamic nuclei, Schizophrenia, Laminar connectivity, Spindle dynamics, Spindle propagation

## Abstract

Sleep spindles are associated with the beginning of deep sleep and memory consolidation and are disrupted in schizophrenia and autism. In primates, distinct core and matrix thalamocortical (TC) circuits regulate sleep spindle activity through communications that are filtered by the inhibitory thalamic reticular nucleus (TRN); however, little is known about typical TC network interactions and the mechanisms that are disrupted in brain disorders. We developed a primate-specific, circuit-based TC computational model with distinct core and matrix loops that can simulate sleep spindles. We implemented novel multilevel cortical and thalamic mixing, and included local thalamic inhibitory interneurons, and direct layer 5 projections of variable density to TRN and thalamus to investigate the functional consequences of different ratios of core and matrix node connectivity contribution to spindle dynamics. Our simulations showed that spindle power in primates can be modulated based on the level of cortical feedback, thalamic inhibition, and engagement of model core versus matrix, with the latter having a greater role in spindle dynamics. The study of the distinct spatial and temporal dynamics of core-, matrix-, and mix-generated sleep spindles establishes a framework to study disruption of TC circuit balance underlying deficits in sleep and attentional gating seen in autism and schizophrenia.

## Author Summary

Sleep spindles are slow brain oscillations that are associated with the beginning of deep sleep, learning, and memory storage and are disrupted in brain disorders. We developed a computational model that can simulate sleep spindles in humans, using novel data on the organization and connectivity of circuits that link the thalamus and cortex, and generate spindles. Our model sheds light on the role of excitatory and inhibitory neurons in the network dynamics and the functional consequences of differential engagement and connectivity of thalamic and cortical areas involved in typical brain function. Our work also establishes a framework for the future study of the dynamics of variable types of sleep spindles and their disruption that can lead to deficits in sleep, memory, and attention.

## INTRODUCTION

Sleep spindles are widespread oscillations associated with the beginning of deep NREM sleep, learning, and memory consolidation (Lüthi, 2014; Steriade, 2005; Steriade, Domich, Oakson, & Deschenes, 1987). Because sleep spindles are disrupted in a variety of neurological and psychiatric conditions, they are an important clinical marker of atypical brain function in several disorders, including schizophrenia and autism (Farmer et al., 2018; Ferrarelli et al., 2007; Manoach et al., 2010; Mylonas et al., 2022).

Reciprocal connections between thalamic nuclei and cortical areas that are gated by the inhibitory thalamic reticular nucleus (TRN) form thalamocortical (TC) circuits that regulate sleep spindle activity (Jones, 2007). However, in mammals two anatomically and functionally distinct TC circuits can be identified, the ‘core’ and the ‘matrix’ loops (Jones, 1998; Müller et al., 2020; Rovo, Ulbert, & Acsady, 2012; Zikopoulos & Barbas, 2007b), and it is not clear how spindle activity and associated functions are regulated by each circuit through synaptic, cellular, or regional interactions at the level of the cortex or thalamus. The core TC circuits, prevalent in sensory thalamus, drive activity focally in the middle cortical layers. In turn, these core thalamic neurons are innervated by small ‘modulatory’ cortical axon terminals from pyramidal neurons in layer 6 (L6). The matrix TC circuits, prevalent in high-order thalamus, have a complementary organization: a mix of large and small axon terminals from cortical layer 5 (L5) pyramidal neurons drive activity of matrix thalamic neurons that, in turn, innervate broadly and modulate the superficial cortical layers. The relative prevalence of the two TC loops differs, with core circuits prevailing in primary and unimodal association cortical areas and first-order relay nuclei, whereas matrix circuits predominate in association and limbic cortical areas and high-order thalamic nuclei (Harris & Shepherd, 2015; Jones, 1998; Rouiller & Welker, 2000; Rovo et al., 2012; Sherman & Guillery, 1996, 2006). However, there is considerable overlap of these parallel loops across TC networks, increasing the complexity of the system (Jones, 1998; Müller et al., 2020; Rovo et al., 2012; Zikopoulos & Barbas, 2007b).

Importantly, in addition to the excitatory connections in the thalamus, both TC circuits likely engage an extensive network of local thalamic inhibitory interneurons that have expanded significantly in evolution and constitute a hallmark of the primate thalamus (Arcelli, Frassoni, Regondi, De Biasi, & Spreafico, 1997), increasing the complexity of potential interactions in primates. Moreover, the connectivity of the inhibitory TRN with core and matrix TC loops is not well studied. The TRN is a key generator of spindle oscillations that intercepts all TC communications, but sends inhibitory projections only back to the thalamus (Lüthi, 2014; Steriade, 2005; Steriade et al., 1987). The TRN gets most of its cortical input from L6 pyramidal neurons that participate in core TC loops (Bourassa & Deschenes, 1995; Bourassa, Pinault, & Deschenes, 1995; Kakei, Na, & Shinoda, 2001), but is predicted to get substantial input from L5 pyramidal neurons of matrix TC loops in primates (Zikopoulos & Barbas, 2006, 2007a), as was recently shown to some extent in mice (Hádinger et al., 2022; Hádinger et al., 2019; Prasad, Carroll, & Sherman, 2020).

Based on this evidence, we set out to test the impact of local thalamic inhibition, variable levels of cortical L6 and L5 pyramidal neuron terminations in TRN, and the effects of these connections in core and matrix TC circuits and spindle activity through the construction of a rate-based computational TC model. Our model successfully simulated relay and filtered signals to sustain and propagate spindle oscillations with different powers, depending on the level of cortical feedback, thalamic inhibition, and involvement of model core versus matrix circuits. Our simulations additionally revealed differences in the spatiotemporal dynamics of core-generated, matrix-generated, or mixed types of spindles, highlighting novel circuit mechanisms involved in typical TC functions, like the sleep–wake cycle, sensory processing, attentional gating, and memory consolidation. Importantly, characterization of novel interactions between key nodes of TC circuits points to potential disruptions of mechanisms that may underlie atypical spindle dynamics in disorders, including autism, and schizophrenia.

## METHODS, MODELS, AND THEORY

### Model Design: Basic Connectivity Frame

**Figure 1.**
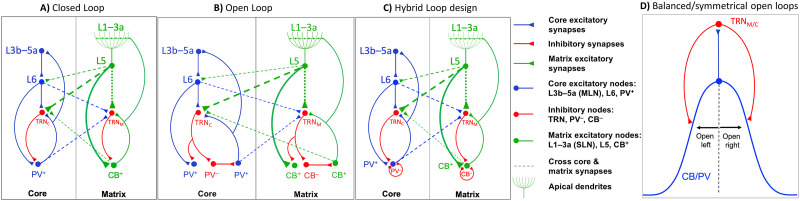
The proposed basic thalamocortical (TC) core, matrix, and mix circuit. The model circuit was based on two parallel thalamocortical loops. Core: PV^+^ excitatory thalamic neurons project focally to the middle cortical layers and get feedback from cortical layer 6. Matrix: CB^+^ thalamic neurons innervate broadly the superficial cortical layers and receive projections from cortical layer 5. Layer 5 terminals in the thalamus are larger (thick arrowheads) than terminals from layer 6. Thalamic and cortical neurons project to TRN (TRN_C_ and TRN_M_). Mix: The two parallel loops cross-connect at the level of the thalamus and/or cortex. The mix dynamics were implemented in three different ways, indicated by dashed lines cross-linking core and matrix loops in panels A–C: (1) *corticoreticular*, where L6 and L5 concurrently excited TRN_M_ and TRN_C_; (2) *thalamoreticular*, where PV^+^ and CB^+^ concurrently excited TRN_M_ and TRN_C_; and (3) cortical, where mixing of core and matrix networks occurred at the level of the cortex, through columnar laminar interactions, simplified as a cross-connection between L5 and L6 (dashed line); this way, we could modify core (L6 and PV^+^) versus matrix (L5 and CB^+^) contribution in the mix. (A) In closed loop connections, TRN neurons directly inhibit the thalamic neurons that excite them. (B) The anatomical circuits underlying open loop connections are shown, in which TRN neurons do not directly inhibit the thalamic neurons that excited them. (C) In the model we simulated the open loop architecture through implementation of the closed loop connections in which TRN neurons directly inhibit the thalamic neurons that excited them, following a symmetric Gaussian spread with the peak at the reciprocal thalamic neuron. Accordingly, the *bilateral* and symmetric Gaussian connectivity strength from TRN to thalamus resembles bilateral and symmetric open loops with opposite open directions (balanced). We called this the Hybrid Loop design. Inhibitory model neurons are shown in red, including TRN (TRN_C_ and TRN_M_ for core and matrix), PV^−^, and CB^−^ local thalamic inhibitory interneurons. The local inhibitory thalamic neurons PV^−^ and CB^−^ send GABAergic projections to thalamic projection neurons (PV^+^ and CB^+^; shown in panels B and C) and can receive excitatory input from the periphery, subcortical nuclei, including TRN, and cortical areas. For simplification of the schematic diagram, the local inhibitory thalamic neurons PV^−^ and CB^−^ in B are shown to receive inhibitory input only from TRN to highlight their potential role in open loop TRN-TC architecture, whereas in C they are shown to receive input from TRN or from cortex and they send inhibitory projections to TC neurons. Blue units are core excitatory elements, that is, neurons in cortical layers 4 and 6 (L3b–5a and L6), as well as thalamic PV^+^ neurons. Green units are matrix excitatory units, that is, neurons in cortical layer 5 (L5), whose apical dendrites reach upper layers (L1–3a) and receive widespread input from excitatory thalamic CB^+^ neurons. Note that in the primate thalamus, PV and CB stain cell bodies and dendrites of core and matrix TC projection neurons, respectively. There is some neuropil staining, especially PV axons and terminals, that come mainly from TRN and basal ganglia; however, the cellular label is predominant and clearly distinguishes the two systems. In several nuclei, for example, the ventral anterior, PV, and CB neurons are intermingled and at equal numbers, so it is not possible to define pure core or matrix regions (Zikopoulos & Barbas, 2007b). (D) When the Gaussian peak of connectivity strength is to the thalamic neuron that directly excites the TRN neuron, due to spatially symmetric bilateral Gaussian spread of connectivity strength, the circuit is composed of two open loops in opposite directions (balanced), then our architecture is functionally similar to a closed loop.

The model is based on key molecular, anatomical, and connectivity features of distinct TC circuits and their specialized interactions with the inhibitory TRN in primates that frame a basic TC circuit, as elaborated below, and summarized in Figure 1.

There are two parallel functionally, structurally, and neurochemically distinct TC loops: the core and matrix (Jones, 2007). Thalamic neurons in core and matrix TC circuits of primates can be distinguished neurochemically (Clasca, Rubio-Garrido, & Jabaudon, 2012; Münkle, Waldvogel, & Faull, 2000). In core loops, excitatory TC projection neurons express the calcium-binding protein parvalbumin (PV). Core PV^+^ excitatory thalamic neurons project focally and drive activity of the middle cortical layers (mainly 4, but also 3b, and 5a) and get feedback from cortical layer 6. In the parallel matrix loops, excitatory TC projection neurons express the calcium-binding protein calbindin (CB). Matrix CB^+^ thalamic neurons innervate broadly and modulate primarily the superficial cortical layers (1–3a), recruiting a broad horizontal spread that targets apical dendrites of pyramidal cortical neurons from layers 2, 3, and 5, which extend to the upper cortical layers. Cortical layer 5 neurons target and drive activity of matrix CB^+^ thalamic neurons.

The entirely inhibitory TRN in primates covers almost the entire thalamus and gates all reciprocal connections between the cortex and the thalamus (Zikopoulos & Barbas, 2007a). The TRN receives input from excitatory thalamic projection neurons and pyramidal neurons in the cortex that project to the thalamus. Anatomical and physiological data suggest that cortical input drives TRN activity (Liu & Jones, 1999; Zhang & Jones, 2004). In turn, the TRN sends inhibitory projections only back to the thalamus. Until recently, it was thought that layer 6, but not layer 5, cortical pyramidal neurons project to TRN (Guillery, 1995, 2005; Guillery, Feig, & Lozsádi, 1998; Guillery & Harting, 2003; Guillery & Sherman, 2002; Kakei et al., 2001; Rouiller & Durif, 2004; Rouiller & Welker, 1991, 2000; Sherman & Guillery, 2002). We first provided strong indirect evidence for a projection from layer 5 to TRN when we studied prefrontal corticothalamic projections and compared them with projections from sensory association cortices and other corticosubcortical projections in primates (Zikopoulos & Barbas, 2006, 2007a). Like other cortices, prefrontal areas project to the thalamus mainly from layer 6, but also issue significant projections from layer 5 (Xiao, Zikopoulos, & Barbas, 2009). We found that prefrontal layer 5 axon terminations in the thalamus constituted a mix of large and small boutons (thin and thick connections in Figure 1), and overall they were larger than terminals from sensory association areas known to originate from layer 6. Recent studies in mice confirmed that there are direct projections of variable density from L5 pyramidal neurons to TRN from some cortical areas, especially association areas that likely participate in matrix circuits (Hádinger et al., 2019, 2022; Prasad et al., 2020). Since the presence of L5 projections to TRN is not ubiquitous, we represent it with a dotted line from L5 to TRN in the simplified diagram in Figure 1, which shows the main nodes of core and matrix circuits.

There is evidence that TRN neurons are paired with thalamic neurons, forming reciprocal, closed loop circuits, in which TRN neurons send GABAergic input to the thalamic neurons that innervate them directly (Figure 1A) (Brown, Taheri, Kenyon, Berger-Wolf, & Llano, 2020; FitzGibbon, Solomon, & Goodchild, 2000; Gentet & Ulrich, 2003; Hale, Sefton, Baur, & Cottee, 1982; Lo & Sherman, 1994; McAlonan, Cavanaugh, & Wurtz, 2006; Pinault, 2004; Pinault & Deschenes, 1998; Sherman & Guillery, 1996; Shosaku, 1986; Steriade, McCormick, & Sejnowski, 1993; Warren, Agmon, & Jones, 1994; Willis, Slater, Gribkova, & Llano, 2015), as reviewed in Jones (2007). In contrast, in open loop circuits, TRN neurons innervate other thalamic neurons that do not directly innervate them (Figure 1B) (Crabtree, 1998; Crabtree, Collingridge, & Isaac, 1998; Crabtree & Isaac, 2002; Kimura, 2014; Lam & Sherman, 2005; Lee, Cruikshank, & Connors, 2010; McAlonan et al., 2006; Pinault & Deschenes, 1998).

In our model circuit we accounted for the TRN-thalamic loop architecture complexity by representing innervation strength with Gaussian curves that have variable spread. We called this architecture Hybrid Loop Design (Figure 1C). When the Gaussian peak of connectivity strength is to the thalamic neuron, which directly excites the TRN neuron due to spatially symmetric bilateral Gaussian spread of connectivity strength, the loop is composed of two open loops in opposite directions (balanced; Figure 1D), then our architecture is functionally similar to a closed loop. Conversely, when the connectivity spread is either spatially asymmetrical or follows a shifted Gaussian, then our circuit architecture resembles an open loop. In addition, in primates, there is extensive presence of local inhibitory neurons in the thalamus, shown as PV^−^ and CB^−^ in Figure 1 (Jones, 2007), which may participate in open loop circuits when innervated by TRN and, in turn, innervate TC neurons. Little is known about the connectivity of local inhibitory thalamic neurons, but studies have shown that they receive input from the sensory periphery, the cortex, and the amygdala and form synaptic triads in the thalamus (Kultas-Ilinsky, Yi, & Ilinsky, 1995; Tai, Yi, Ilinsky, & Kultas-Ilinsky, 1995; Timbie, Garcia-Cabezas, Zikopoulos, & Barbas, 2020), as reviewed in Jones (2007).

The two parallel core and matrix circuits are not necessarily isolated, but can overlap across TC networks. This overlap can be extensive in high-order TC networks, like the ones linking the mediodorsal nucleus (MD) or the ventral anterior nucleus with the prefrontal cortex (Jones, 1998; Müller et al., 2020; Rovo et al., 2012; Zikopoulos & Barbas, 2007b). However, it is not known whether this overlap is limited at the level of the thalamus, or if it is also present at the level of the cortex and TRN. To account for the potential overlap of core and matrix circuits at each TC node, we included cross-connections (dashed lines in Figure 1) that can facilitate *mixing* of the two parallel loops at all levels. For cortical core contribution in the mix, L6 directly projects to TRN_M_, and for cortical matrix contribution in the mixing, L5 directly projects to TRN_C_. For thalamic core contribution in the mix, PV^+^ directly projects to TRN_M_, and for thalamic matrix contribution in the mix, CB^+^ directly projects to TRN_C_. We also include mixing of core and matrix networks at the level of the cortex through several laminar interactions, simplified in Figure 1 as a cross-connection between L5 and L6 (dashed line). The source codes for the neural model are available in the Supporting Information.

### Mathematical Specification of the Rate Model: Dynamics of Model Neurons

We constructed a computational model of core and matrix TC circuits based on the connectivity above. We expressed each simulated neuron’s activity, based on inhibitory and excitatory inputs, over time, as a first-order differential equation. Model neurons were represented by a single-compartment voltage (*v*) over time (Layton, Mingolla, & Yazdanbakhsh, 2012, 2014; Layton & Yazdanbakhsh, 2015) that obeys the following shunting equation:

$$\frac{dv}{dt}=-Av-\left(v-E_{\mathrm{ext}}\right)I_{\mathrm{ext}}-\left(v-E_{inh}\right)I_{inh}$$ (1)

In this equation, *A* denotes the constant decay (leakage) rate, which brings *v* back to zero when there is no excitatory or inhibitory input to the neuron to fulfill the physiological constraint that a neuron without input finally goes back to its resting potential. *I*_*ext*_ and *I*_*inh*_ specify the summed excitatory and inhibitory inputs to the neuron at each time. The terms *E*_*ext*_ and *E*_*inh*_ refer to excitatory and inhibitory reversal potentials, respectively, which keep *v* within *E*_*ext*_ and *E*_*inh*_ range to fulfill the physiological constraint of the limited dynamical range of each neuron activity. We set *E*_*ext*_ and *E*_*inh*_ equal to 1 and −1; therefore, our model neurons activities fell within [−1, 1] range. Table 1 shows *I*_*ext*_ and *I*_*inh*_ for each model neuron type as illustrated in Figure 1.

**Table 1.** Model neurons and their excitatory and inhibitory inputs (see Figure 1)

**Model neuron**	**Symbol**	**Excitatory input (*I*_*ext*_)**	**Inhibitory input (*I*_*inh*_)**
Matrix excitatory thalamic neurons	${CB}_{i}^{+}$	$\sum_{j=1}^{N}{\left(L_{5}\to {CB}^{+}\right)}_{ji}L_{5}^{j}$	$\sum_{j=1}^{N}{\left({CB}^{-}\to {CB}^{+}\right)}_{ji}CB_{j-\eta }^{-}+{\left({TRN}_{M}\to {CB}^{+}\right)}_{ji}{TRN}_{M}^{j}$
Core excitatory thalamic neurons	${PV}_{i}^{+}$	$\sum_{j=1}^{N}{\left(L_{6}\to {PV}^{+}\right)}_{ji}L_{6}^{j}$	$\sum_{j=1}^{N}{\left({PV}^{-}\to {PV}^{+}\right)}_{ji}PV_{j-\eta }^{-}+{\left({TRN}_{C}\to {PV}^{+}\right)}_{ji}{TRN}_{C}^{j}$
Matrix inhibitory thalamic neurons	${CB}_{i}^{-}$	$\sum_{j=1}^{N}{\left({CB}^{+}\to {CB}^{-}\right)}_{ji}CB_{j}^{+}$	$\sum_{j=1}^{N}{\left({TRN}_{M}\to {CB}^{-}\right)}_{ji}{TRN}_{M}^{j}$
Matrix inhibitory thalamic neurons	${PV}_{i}^{-}$	$\sum_{j=1}^{N}{\left({PV}^{+}\to {PV}^{-}\right)}_{ji}PV_{j}^{+}$	$\sum_{j=1}^{N}{\left({TRN}_{C}\to {PV}^{-}\right)}_{ji}{TRN}_{C}^{j}$
TRN matrix	${TRN}_{M}^{i}$	$\sum_{j=1}^{N}{\left({CB}^{+}\to {TRN}_{M}\right)}_{ji}{CB}_{j+\eta }^{+}+{\left(L_{5}\to {TRN}_{M}\right)}_{ji}L_{5}^{j}$	$\sum_{j=1}^{N}{\left({TRN}_{M}\to {TRN}_{M}\right)}_{ji}TRN_{M}^{j}$
TRN core	${TRN}_{C}^{i}$	$\sum_{j=1}^{N}{\left({PV}^{+}\to {TRN}_{C}\right)}_{ji}{PV}_{j+\eta }^{+}+{\left(L_{6}\to {TRN}_{C}\right)}_{ji}L_{6}^{j}$	$\sum_{j=1}^{N}{\left({TRN}_{C}\to {TRN}_{C}\right)}_{ji}TRN_{C}^{j}$
Superficial layer neurons (SLN)	$L_{1-3a}^{i}$	$\sum_{j=1}^{N}{\left({CB}^{+}\to L_{1-3a}\right)}_{ji}CB_{j+\eta }^{+}$	
Middle layer neurons (MLN)	$L_{3b-4}^{i}$	$\sum_{j=1}^{N}{\left({PV}^{+}\to L_{3b-4}\right)}_{ji}PV_{j+\eta }^{+}$	
Cortical layer 5 neurons	$L_{5}^{i}$	$\sum_{j=1}^{N}{\left(L_{1-3a}\to L_{5}\right)}_{ji}L_{1-3a}^{j}$	
Cortical layer 6 neurons	$L_{6}^{i}$	$\sum_{j=1}^{N}{\left(L_{3b-4}\to L_{6}\right)}_{ji}L_{3b-4}^{j}$	

*Note*. *η* = offset to simulate open loop (see Figure 1); → = intercortical, thalamocortical, thalamoreticular, or corticoreticular connection.

To facilitate description of excitatory/inhibitory interactions (*I*_*ext*_ and *I*_*inh*_), we constructed thalamic, reticular, and cortical networks as a distance-dependent on-center- off-surround shunting network, as we have described in the past (Layton et al., 2012, 2014; Layton & Yazdanbakhsh, 2015; Sherbakov & Yazdanbakhsh, 2013; Wurbs, Mingolla, & Yazdanbakhsh, 2013) and recently in computational models of cortico-thalamic-amygdalar circuits (John, Bullock, Zikopoulos, & Barbas, 2013; John, Zikopoulos, Bullock, & Barbas, 2016). Such distant-dependent on-center interareal interactions are modeled by bell-shaped (Gaussian) profiles reflected in *I*_*ext*_ column of Table 1 by (*X* ➔ *Y*) symbols, in which *X* and *Y* indicate the presynaptic and postsynaptic regions, for example, in row 1 of Table 1, *L*_5_ and *CB*^+^, respectively. Similarly, the off-surround distant dependent reflected in the *I*_*inh*_ column. Networks of this type offer a simple, biologically plausible means to implement contrast enhancement and a host of other processes by varying the strength of the off-surround inhibition to modulate the tuning curve of each cell (Cao, Mingolla, & Yazdanbakhsh, 2015; Qian & Yazdanbakhsh, 2015). Strong inhibition yields sharp contrast, fine tuning, and high attention. Weak inhibition leads to spreading activity, lower contrast, and inattentiveness. These processes may be altered in autism and schizophrenia, leading to deficits in attentional gating (Medalla & Barbas, 2009). Thus, modulating the balance between excitation and inhibition in various nodes of the circuit can provide a way to investigate the effects of various network elements on signal processing and attention.

In the model, we took into account both anatomical (connectivity) and physiological differences in L5 and L6 neurons and their terminals. In our simulations, these differences were reflected by the parameterization of the model, so that the L5 ➔ CB connection was set to be 20 times (∼1 order of magnitude) stronger than L6 ➔ PV. This is in line with physiology data suggesting more burst firing and larger excitatory postsynaptic potentials with more paired pulse depression for L5 neurons and terminals (Agmon & Connors, 1992; Chagnac-Amitai, Luhmann, & Prince, 1990; de Kock et al., 2021; Greenhill, Ranson, & Fox, 2015; Larkum, Zhu, & Sakmann, 1999; Livingstone, Freeman, & Hubel, 1996; Llano & Sherman, 2009; Shai, Anastassiou, Larkum, & Koch, 2015), but also with the anatomy, which has shown that L5 terminals in the thalamus are much larger than L6 terminals and can drive CB neuron activity (Bourassa et al., 1995; Hoogland, Wouterlood, Welker, & Van der Loos, 1991; Llano & Sherman, 2009; Zikopoulos & Barbas, 2007b). Varying the relative strength of L5 ➔ CB connection changed the oscillatory tendency of the matrix loop (shown in the Results).

### Equations for Model Neurons

Equation 1 is the general equation for all neurons of the model circuit of Figure 1. The key distinction between model neurons is based on their excitatory (*I*_*ext*_) and inhibitory (*I*_*inh*_) inputs stemming from the neuroanatomy of the connectivity; therefore, the current neural model reflects the impact of neuroanatomy in generating distinct spatiotemporal dynamics of core and matrix TC loop spindles (Piantoni, Halgren, & Cash, 2016), based on the position and distribution of neurons rather than differences of reticular, thalamic, and cortical neuron types. Each row of Table 1 represents the presynaptic total excitatory and inhibitory input to each model neuron (i.e., CB^+^, TRN_M_, etc.). Indices *i* and *j* indicate the spatial position of model neurons *i* and *j* on a one-dimensional array. Pre- to postsynaptic connections are characterized by (Pre ➔ Post) symbols subindexed by *ji* indicating the *j*th presynaptic to *i*th postsynaptic neuron strength. The relative strength of *j*th presynaptic to *i*th postsynaptic neuron follows a normal distribution centered at *i* (i.e., the *i*th pre- to *i*th postsynaptic connection is the strongest).

$${\left(pre\to \text{post}\right)}_{ji}=\frac{1}{\sigma \sqrt{2\pi }}e^{-\frac{1}{2}{\left(\frac{j-i}{\sigma }\right)}^{2}}$$ (2)

### Spindles in the Model TRN

**Figure 2.**
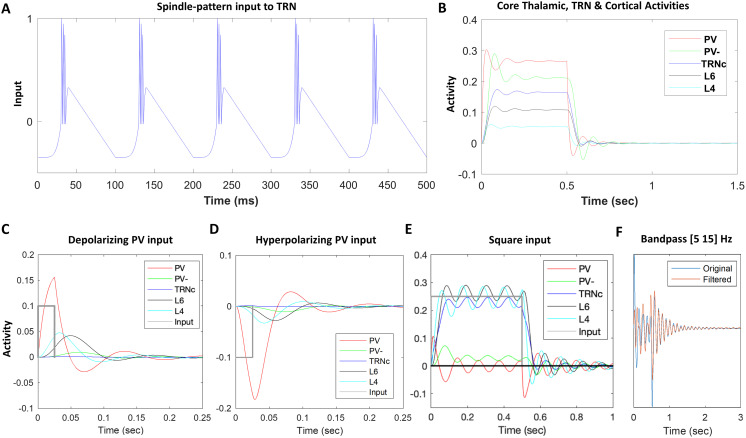
Model inputs and response. (A) Model TRN input resembling spindle burst temporal pattern. Spindle generation in the model is based on inducing TRN neurons by inputs resembling the spindle spike temporal patterns, which can entrain the model thalamocortical loop depending on the tendency of rebound depolarization and oscillation. Throughout the reported simulations, we chose a 10 Hz (100-ms interburst interval) spindle-pattern input with a 500-ms duration to induce the model TRN units. To set the TRN spindle inducing pattern as similar to a physiological spindle as possible, we considered physiological properties of TRN neurons (reviewed in McCormick & Bal, 1997) and approximated the initial membrane potential raise and then an accelerated upturn (T-current) before the spikes burst by a sixth-degree polynomial. We also approximated the bursts by densely packed spike-like pattern between 0 and 1 to have the model neuron activities within the normalized values throughout the simulation. (B) Model response to 0.5-sec tonic input to PV. Each layer neuron activity is amplitude scaled to prevent overlap for clear visibility. During the input onset, each model neuron depolarizes, reaches its peak, reverses, and after a few oscillations settles. After turning off the square input at 0.5 sec, the activities drop sharply toward baseline followed by hyperpolarization, rebound depolarization, and oscillations to finally settle on the resting state. Response to a 0.5-sec square input illustrates the dynamics of the rate-based model and its tendency for rebound depolarization. (C) Model response to a depolarizing input pulse. After PV depolarization, there is hyperpolarization and a rebound depolarization. (D) Model response to a hyperpolarizing input (due to a brief input pulse to TRN). PV neurons exhibit hyperpolarization first and then they rebound depolarize. (E) Model response to 0.5-sec depolarizing tonic input, while the network is in high spindle tendency, due to increased levels of TC inhibition by TRN, shows oscillatory activity of higher amplitude and longer duration compared to the response in panel B with the network in lower spindle tendency; here too, each layer neuron activity is amplitude scaled to highlight PV oscillations. (F) Band-pass filtering typically used to filter the signal in the relevant frequency bands; in this example, 5–15 Hz produces output similar to the original model output.

Neurons in the TRN send inhibitory GABAergic projections to thalamus (Figure 1). TRN neurons can fire in bursts, generating inhibitory postsynaptic potentials (IPSPs) in excitatory thalamic neurons (PV^+^ and CB^+^), which in turn exhibit rebound bursts, activating the TRN neurons. By simulating neurons using the rate-based approach detailed above, we first introduced burst-like activity input in our model TRN neurons (Figure 2A), which mimics the temporal dynamic of constituent bursts in a spindle. The *y*-axis in Figure 2A shows normalized values with the lower and upper limits of −1 and +1, similar to the lower and upper bounds of all of the model neurons’ activities. The interburst interval was set to 100 ms to replicate a 10 Hz burst rate in 0.5 sec. Such a voltage induction in model neurons is within the physiological range of sleep spindles with a frequency of 7–15 Hz and duration of 0.5–3 sec (Lüthi, 2014; Steriade, 2005; Steriade et al., 1987). In order to have the input temporal dynamics of Figure 2A as close as possible to the burst sequence of spindles, we approximated physiological voltage patterns shown in previous work (McCormick & Bal, 1997) by a sixth-degree polynomial to offer a slow initial increase and then a sharp raise mimicking T-current before the burst spikes. After this stage, the burst is approximated by a densely packed spike-like pattern between 0 and 1. For visibility, the burst spike peaks are slightly decreasing sequentially with no effect on the model activity dynamics. We therefore approximated the activity of TRN neurons recorded intracellularly, in vivo, which communicate with each other through GABA**_A_** receptor-mediated synapses, inducing chloride ion channel-mediated IPSPs. The chloride reversal potential is approximately −71 mV, which is relatively depolarized compared to the −78 mV resting membrane potential of TRN neurons resulting in IPSP-triggered low-threshold spikes (LTSs) (Bazhenov, Timofeev, Steriade, & Sejnowski, 1999). We approximated the −78 to −71 mV dynamic leading to LTS by a sixth-degree polynomial (slow to fast upward curving up before burst in Figure 2A).

Through injection of spindle-pattern input in TRN, we bypassed sleep spindle generation in the TRN and focused on the examination of the dynamics of core and matrix loops, in terms of oscillatory activity and spindle tendency in the cortex and thalamus. However, since the model, based on its architecture, could oscillate independently, we additionally provided the model response to tonic depolarizing, hyperpolarizing, or 0.5-sec square inputs to show that the model also generates spindle-like oscillation in response to tonic inputs (Figure 2C–E). We used this approach, to additionally test oscillatory activity and spindle tendency in our simulations.

### TRN Induced IPSPs in TC Neurons and Rebound

In the model circuit of Figure 1, TRN neurons project to the excitatory thalamic neurons in core (PV^+^) and matrix (CB^+^) by inhibitory connections (representing GABA synapses). When model TRN neurons receive the spindle inducing input (Figure 2A), they generate IPSPs in TC neurons (Lüthi, 2014; McCormick & Bal, 1997). The postsynaptic potential in TC neurons induces a hyperpolarizing H-current leading to cation low-threshold Ca^2+^ channel T-currents before the rebound bursts. The bursts of TC neurons activate the TRN neurons through the excitatory synapses of the model circuit (Figure 1; representing glutamatergic input to glutamate receptors of TRN neurons). Figure 2B shows the mutual interaction of model TRN and TC (i.e., PV^+^) neurons induces rebound activity of TC neurons after hyperpolarization (see, e.g., red PV^+^ curve right after 0.5 sec). The rebound in the simple compartment rate-based model stems from the decay term −*Av* in Equation 1 which brings the hyperpolarized and/or depolarized *v* back to zero with the rate *A* combined with the excitatory and inhibitory neurons’ interactions in the model circuit. Although the simplified mechanisms in the model are by far less sophisticated than the actual physiology of membrane voltage modulation through the variety of hyperpolarization activated cationic H-currents, low-threshold Ca^2+^ channels T-current, neurotransmitter gated and voltage gated channels, they nevertheless, approximate the hyperpolarization and rebound of TC neurons.

### Data Analysis

Figure 2B illustrates oscillation tendency of the model core thalamocortical loop; after the input is discontinued, activity drops sharply to rest, further reduces toward hyperpolarization, after which activity rebounds toward depolarization. Such a dynamic can cycle a few more times, and more cycles indicate more rebound depolarizations after input shutdown, which are substrates for spindle sustaining. Therefore, we define spindle tendency index (*STI*) as the total duration (in seconds) of the sequence of depolarization rebounds after hyperpolarization. We consider 1% of maximum activity level of a model neuron as the threshold for the peak of a depolarization to be counted as a rebound for inclusion within the hyperpolarization-depolarization duration after input shutdown. The 1% thresholding stemmed from the minor, subthreshold depolarization needed to activate the H-current in neurons in vivo to initially and partially depolarize the membrane before the T-current taking over and leading to bursting. Changing the threshold did not change the ordinal relations of spindle tendencies across conditions. We considered the unit of time in seconds because average spindle duration is ∼1 sec, and therefore the number of seconds conveys how many times of an average spindle duration a hyperpolarization-depolarization sequence lasts as an indicator of spindle tendency. For example, in Figure 2B, the duration of the above threshold hyperpolarization-depolarization sequence after the input shutdown is 0.37 sec; hence, *STI* = 0.37. The index enables us to compare the tendency of model core, matrix, and mix TC circuits to sustain spindles, as reported in the Results section. We additionally checked for oscillatory spindle tendency through filtering of signals within the relevant frequency band being thresholded (band-pass filtering 5–15 Hz; Figure 2F).

Moreover, we visualized the spatiotemporal characteristics of core, matrix, and mixed TC circuits using heat map matrices, and, to facilitate comparisons, we calculated dot products of vectorized matrices and then normalized the dot product by the absolute value of each vector, that is, A→·B→AB, in which $\vec{A}$ and $\vec{B}$ are vectorized heat map matrices and |*A*| and |*B*| are their size (scalar). This normalized value is between −1 and 1 (in all of our cases between 0 and 1); closer to 1 means more similarity (parallel) between $\vec{A}$ and $\vec{B}$ and closer to 0 means more dissimilarity (orthogonal). In order to show the similarity and difference with a finer grain, we calculated the *ArcCos* of cosine similarity, that is, ***θ*** = *ArcCos* (A→·B→AB) to obtain the angle ***θ*** between spatiotemporal patterns. ***θ*** = 0 means complete similarity (parallel), and the more ***θ*** deviates from zero the similarity decreases.

## RESULTS

### Rate-Based Model Simulations: Ability to Sustain and Propagate Activity

We constructed a computational TC circuit that included core and matrix components with an optional and variable cortical L5 to TRN projection (L5-TRN ON/OFF). Based on the features of TC circuits, our model was able to simulate relay and filtering of signals and could propagate and sustain spindle oscillations. As such, throughout the simulation results reported in the following sections, the state of the network supporting the spindle was based on the presence and amplitude of single *vs* sustained oscillations. In addition, parameterization of the model, so that the L5 ➔ CB connection was set to be 20 times (∼1 order of magnitude) stronger than L6 ➔ PV to reflect anatomical (connectivity) and physiological differences in L5 and L6 neurons and their terminals, appeared to be necessary to keep the oscillatory tendency of the matrix loop. This is due to major connectivity pattern differences of the matrix loop, which has spatially broader modulatory input to the upper layers of the cortex, compared to the spatially narrower, feedforward driving input of the core TC in the middle layers. Two orders of magnitude stronger L5 ➔ CB connection compared to L6 ➔ PV also worked, showing a wider connectivity strength tolerance for the matrix loop. The limit in our rate-based model was 3 orders of magnitude in which matrix lost its oscillatory tendency.

To evaluate the readiness of the network to generate or maintain spindles, we gave the model TRN neurons of the model core TRNc the spindle-like input depicted in Figure 2 for 500 ms with starting time at 0 reflected on the *x-*axis. As indicated in Figure 2 and elaborated in the Data Analysis section, we shut down the spindle-like input at time 0.5 sec. After the spindle input induction, we left the network on its own, to see how the model TC loop could sustain postspindle oscillations (sequential hyperpolarizations and depolarization rebounds) to estimate the STI. STI is a relative measure of network spindle tendency that can be used to compare the model core, matrix, and mix in supporting spindle generation and sustaining. Using STI, we can also compare model spindle tendency with and without layer 5 corticothalamic projections to TRN, local thalamic inhibition, and with mixing of parallel loops. We additionally checked for oscillatory spindle tendency through band-pass filtering (5–15 Hz; Figure 2F). The result of the band-pass filtering metric was consistent with the STI metric, because the rate-based model in response to tonic or oscillatory inputs generated regular and consistent frequency. Therefore, the band-pass filtering output and the original output were consistent and power thresholding was the same (Figure 2F).

The simulated oscillatory activity was subthreshold (Figure 2B–E) and in the case of TC neurons resembled minor rebound depolarizations that can be considered as an indicator/marker of a burst (McCormick & Bal, 1997; McCormick & Pape, 1990). This is because due to the nature of rate-based models, a burst cannot be directly generated and visualized (there is no TC sequence of H-current and T-current built into rate-based models). Instead, a minor subthreshold rebound depolarization is an indicator of the tendency of the network to generate a burst and the subthreshold oscillatory activity after the initial input is an indicator of spindle oscillations. This is supported by previous physiological work that showed the subthreshold pacemaker potential of TC neurons, demonstrating that a TC neuron burst starts with a minor subthreshold depolarization with H-current activation, which slightly depolarizes the neuron (Deschenes, Paradis, Roy, & Steriade, 1984; McCormick & Bal, 1997; McCormick & Pape, 1990; Steriade & Deschenes, 1984). After this initial phase, the T-current then takes over and pushes the membrane voltage from its minor initial depolarization further up to cross the threshold leading to an action potential followed by a burst.

Moreover, as can be seen in Figure 2, the model has an oscillatory tendency with similar general features, regardless of the input: (a) in response to a depolarizing input pulse to TC neurons (Figure 2C), where after PV depolarization, there is hyperpolarization and a rebound depolarization, or (b) in response to a hyperpolarizing input on TC neurons (due to a brief input pulse to TRN), where after PV hyperpolarization, there is rebound depolarization (Figure 2D). Use of a tonic depolarizing input to TC neurons instead of the initial oscillatory input to TRN for simulations did not change the model dynamics and findings of the study: injecting a tonic input of longer duration (0.5 sec), as shown in Figure 2E, led to high oscillatory activity and high spindle tendency.

### Impact of L5 to TRN_M_ Connections

**Figure 3.**
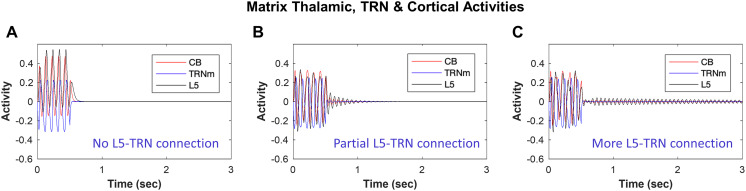
The effect of model TRN input resembles spindle temporal pattern. The model is based on direct and indirect evidence for the presence of L5 neuron projections to TRN_M_. (A) No direct L5 to TRN_M_ results in STI < 0.1, which does not promote spindle tendency in the network. (B) In comparison, implementation of partial, small L5 to TRN_M_ connection (equivalent to 5% of L6 to TRN_**C**_ model connection strength) yields STI = 0.57, which shows a facilitating effect of direct (L5 to TRN_M_) connection in promoting spindle tendency of the network. (C) the efficacy of L5-TRN connection is further increased from 5% to 10% resulting in STI > 1 that can contribute to the elongation of spindling time and can facilitate continuous or repetitive spindles instead of isolated ones.

Projections from L5 to TRN have only recently been shown for some cortical areas and at variable levels (e.g., Prasad et al., 2020; Zikopoulos & Barbas, 2006), with L6 projections to TRN being considered the norm, and several studies showing absence of direct L5 synaptic terminals in TRN (e.g., Bourassa & Deschenes, 1995). This prompted us to investigate the effects of direct L5 to TRN_M_ projection on spindle dynamics. Figure 3A shows the spindle dynamics in matrix TC loop when the connection between model L5 and TRN_M_ is absent. We considered this condition the baseline. In Figure 3B and C, we turned on synaptic connections between L5 and TRN_M_ at a level equivalent of 5–10% of L6-to-TRN_**C**_ model connection strengths. Compared to Figure 3A, the single-compartment voltage dynamics of matrix TC loop model neurons in Figure 3B and C showed a temporal extension of hyperpolarization/depolarization beyond 0.5 sec (when the spindle-like input to TRN was shut down) that gradually increased with increased strength of L5 to TRN_M_ input (from Figure 3B to 3C). This suggests that the presence of direct input from L5 to TRN promotes temporal sustaining of spindles.

### Spindle Wave Propagation

**Figure 4.**
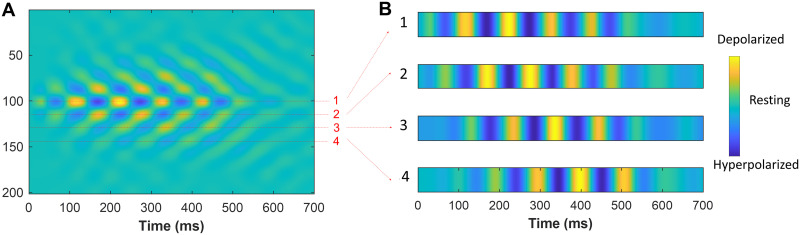
Spindle wave propagation across model neuron arrays. (A) The activity of 200 model TC neurons (PV^+^) over time. Yellow, depolarization; green, resting potential; and blue, hyperpolarization. Model recording sites are indicated by 1, 2, 3, and 4 corresponding to neurons 100, 113, 128, and 147, respectively. (B) From top to bottom, four recording locations 1–4 from the model neurons are shown. Similar to the Kim et al. (1995) recording simultaneously from multiple sites in the ferret LGNd, spindle oscillations propagate across locations 1–4 in the model. Each subpanel of panel B is normalized within its range, and therefore the depolarization and hyperpolarization colors are identical, merely showing whether the membrane potential is depolarized or hyperpolarized. Therefore, the aim of panels 1–4 is to indicate the voltage change direction either toward depolarization or hyperpolarization independent of the color map magnitude, which was adjusted to enhance visibility.

Kim, Bal, and McCormick (1995) recorded simultaneously from multiple sites in the ferret dorsolateral geniculate nucleus (LGNd) in vitro and found that spindle oscillations propagate across the slice. The hyperpolarization and depolarization waves in Figure 4A show the same trend; spindling tendency propagated across locations where model array neurons numbered from 1–200 (*y*-axes) are distributed. Figure 4B shows the hyperpolarization-depolarization dynamics of model TC neurons for four locations indicated in Figure 4A: first being center (neuron no. 100), second location (neuron no. 113), third location (neuron no. 128), and fourth location (neuron no. 147). Following first to fourth locations in order shows that the initiation of spindle tendency propagated from the first to the fourth location. This provided a mechanistic platform to further investigate the propagation dynamics based on the involvement of different constituent parts of the TC loop. The example in Figure 4B is based on a mixed core-matrix circuit with only core (PV^+^) neurons’ membrane potentials shown.

### Impact of Thalamic Local Inhibition on Spindle Dynamics

**Figure 5.**
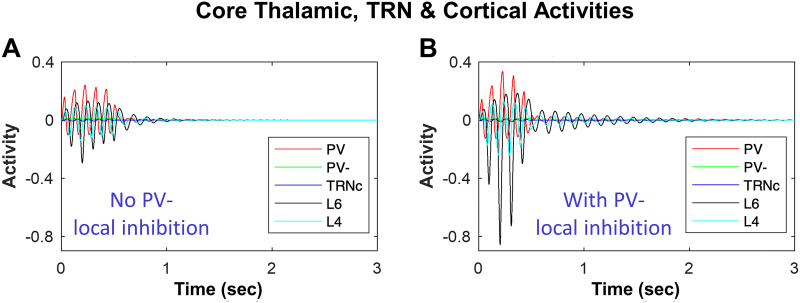
Impact of local thalamic inhibition in biasing the network toward sustaining or terminating spindles. (A) shows the dynamics of core model neurons with the absence of local inhibition (no PV^−^ inhibition of PV^+^), and (B) with the presence of local inhibition. The spindle-like input (shown in Figure 2) is ON for 0.5 sec starting at time 0 and turned OFF at time 0.5 sec. By tracing the behavior of the model TC neurons, we evaluate the tendency of the network to sustain or terminate spindles. Tracing the activity of PV and L4 neurons in panel A and B on and after 0.5 sec indicates longer duration and higher amplitude of hyperpolarization and rebound polarization in panel B compared to A, indicating a higher spindle probability in the model neurons of TC loop with the presence of local inhibition in thalamus.

By tracing the behavior of the model TC neurons under different conditions, we evaluated the tendency of the network to sustain or terminate spindles. Figure 5A shows the model core neuron activities when there is no local inhibition in the thalamus (no PV^−^ ➔ PV). Figure 5B shows the effects of local inhibition in the thalamus on the oscillatory TC activity. The presence of local inhibition in thalamus indicates a higher spindle probability in the model neurons of core TC loop, as seen in the comparison of the traces of activity of PV and L4 neurons on and after 0.5 sec, which lead to STI >1 in Figure 5B compared to STI = 0.55 in Figure 5A. Similarly, local inhibition (CB^−^) in the matrix TC loop increased the spindle probability when the L5-TRN_M_ connection was present. With no L5-TRN_M_ (STI < 0.1; Figure 3A), presence or absence of local inhibition did not make a difference and STI remained <0.1, indicating the necessity of L5-TRN_M_ connection for the gating effect of local inhibition in promoting spindling tendency.

### Impact of TRN Inhibition of Thalamus on Spindle Dynamics

**Figure 6.**
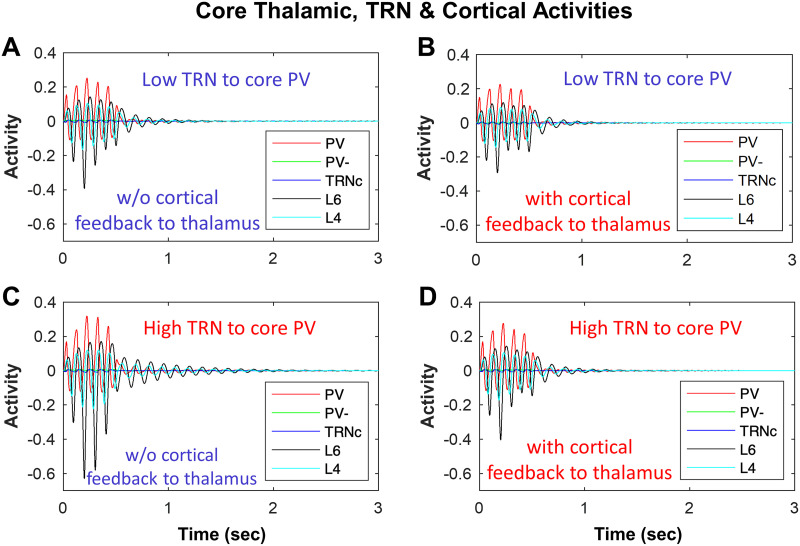
Impact of TRN inhibition and cortical feedback to thalamus in biasing the network toward sustaining or terminating spindles. (A) The dynamics of core model neurons without cortical feedback to thalamus (STI = 0.55) compared to (B) with cortical feedback to thalamus (STI < 0.55), indicating a decreased spindling tendency. (C) Similar to panel A, but with increased TRN inhibition of PV, which increased STI to >1 compared to panel A with STI = 0.55; (D) Similar to panel C, but with cortical feedback to thalamus, which reduced STI to 0.55 from >1 in C.

Following the same approach, we tested the effect of different levels of TRN inhibition and cortical feedback on the TC loop tendency to generate and sustain spindles (Figure 6A–D). Low versus high levels of TRNc input to model excitatory core PV thalamic neurons (Figure 6A–C) showed that during the initial spindle-like input to model TRNc (as shown in Figure 2) from time 0–0.5 sec, the amplitude of hyperpolarization and rebound in core model neurons were higher with increased TRNc inhibition of PV neurons. After the spindle-like input to model TRNc was shut down (after 0.5 sec), the sequence of hyperpolarization and rebound events due to model core TC loop sustained longer over time with higher TRNc inhibition of PV neurons (Figure 6C with STI >1) compared to lower TRN inhibition (Figure 6A with STI = 0.55). A similar trend was present for matrix TC loops; higher TRN_M_ input to excitatory thalamic neurons (CB^+^) increased the STI.

### Impact of Cortical Input to Thalamus on Spindle Dynamics

Corticothalamic feedback in the TC loop controls sleep spindle duration in vivo (Bonjean et al., 2011). In line with this, cortical feedback in our model was a key player in the spindling time and tendency to keep spindles unitary, repetitive, or sustained. In Figure 6A and C we illustrate an example of the dependence of spindle dynamics on synaptic interactions within the thalamoreticular loop, without any cortical feedback. Comparison of Figure 6C (STI > 1) with 6D (STI = 0.55) can provide an example of the impact of model cortex feedback to TC neurons. We found that the effect of cortex was toward reducing the spindling time, and after the spindle-like input was shut down at 500 ms, there was less tendency for spindles, in the form of fewer hyperpolarization and rebound depolarization events. Cortical feedback can be channeled in two forms: cortico-thalamic (shown here in Figure 6, L6 ➔ PV) and corticoreticular (not shown). Consistent with what Bonjean et al. (2011) have shown (see their Figure 4), we also found that in our single-compartment voltage rate-based model, more feedback from L6 to PV (cortico-thalamic) reduces the spindle duration (Figure 6D; compare with 6C with no cortical feedback). On the other hand, greater cortical feedback to TRN in our model leads to greater input from TRN to thalamus and has the opposite effect, increasing spindle tendency, in line with previous findings (Bonjean et al., 2011). The effects of the model matrix cortical feedback to CB and TRN_M_ were similar to the effects observed in the model core.

### The Impact of Mixed Core and Matrix Circuits on Spindle Spatiotemporal Dynamics

**Figure 7.**
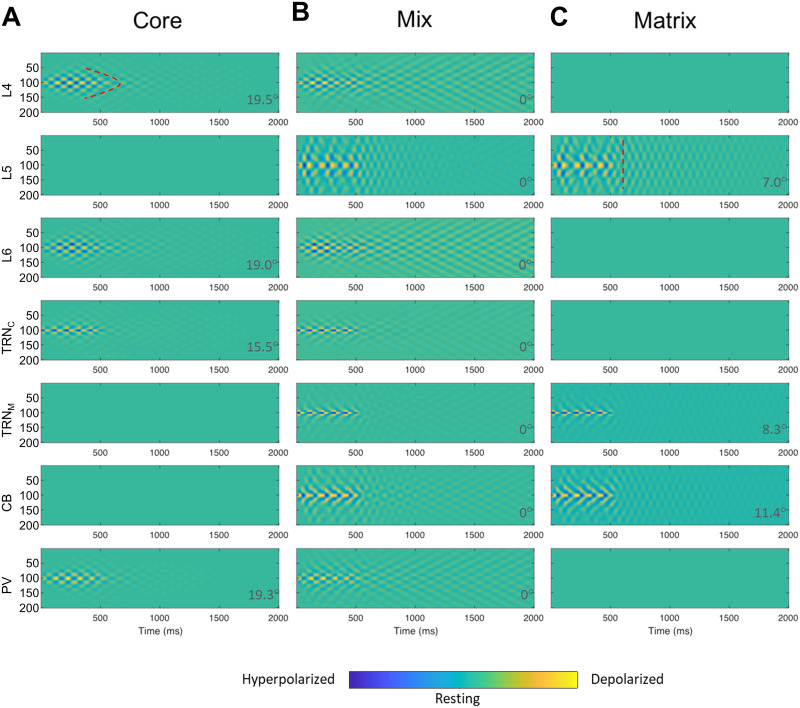
Core, Matrix, and mixed TC model spatiotemporal dynamics. (A) Only model L6, TRN_C_, and PV (core TC loop) were involved in spindle entrainment, induced by inputting spindle-like input (Figure 2) to TRN_C_, while L5, TRN_M_, and CB (matrix) remained inactive. (B) The mix (50–50% thalamoreticular mixing) of core and matrix, engaged all model areas. (C) Only model L5, TRN_M_, and CB (matrix TC loop) were involved in spindle entrainment, induced by inputting spindle-like input (Figure 2) to TRN_M_, while L6, TRN_C_, and PV (core) remained inactive. Spindle-like input (Figure 2), was delivered to neuron no. 100 (*y*-axis) in the TRN_C_ and TRN_M_ array of model neurons, lasting for 500 ms and was then shut down. The presence of color map ripples in the figure panels are equivalent to sequences of hyperpolarization and depolarization events shown in previous figures and indicate spindle tendency of the network (beyond 500 ms based on the TC model dynamics). Comparison of corresponding panels in A and C revealed a broader spatial spread and temporal extent in matrix than core. Notably, the mix circuit spatiotemporal dynamics of spindles in each site was a hybrid of core and matrix spindles in terms of broadness of spatial smear and temporal extent. In panel A, the model core cortical layer 4, L4, showed a focal tendency for spindle sustaining indicated by a curved dotted marking. Compare with panel C model matrix cortical layer 5 (L5), in which the spindle tendency across model neurons after propagation appeared synchronized and therefore the depolarization iso-amplitude traces aligned vertically at each time bin after spindle propagation for a few hundred milliseconds. A vertical dotted mark as an example is shown in panel C, L5. To further compare the effects of mixing, we set 50–50% mixing level in panel B as the baseline (angle ***θ***: *ArcCos* of cosine similarity = 0°). Comparison of ***θ*** values in panel B with pure core in A and pure matrix in C provides an estimate of the blending effects, expressed as rising deviation of the angle ***θ*** from 0°. Higher deviation of the angle ***θ*** from 0° indicated increased dissimilarity from B.

Most TC loops in primate brains appear to include a mixture of core and matrix components (Jones, 2007; Müller et al., 2020; Piantoni et al., 2016; Zikopoulos & Barbas, 2007b). For this reason, we compared the spatiotemporal dynamics of spindles in the mix circuit with those of isolated core and matrix TC circuits (Figure 7). There are multiple ways of mixing core and matrix circuits, namely, through thalamus, cortex, or both. In thalamic mixing (Figure 7), we connected model PV and CB to TRN_M_ and TRN_C_, respectively. In cortico-thalamic mixing (not shown), we connected model L6 and L5 to TRN_M_ and TRN_C,_ respectively. Finally, we also included in the model laminar interactions occurring at the cortical level, which constitute the most widely inferred, modeled, and studied mixing of core and matrix TC circuits. The mixed spindle spatiotemporal dynamics turned out to be a hybrid of the core and matrix spindles in all cases. These results highlight the impact of circuit connectivity on the core and matrix spindle patterns (Piantoni et al., 2016), which blended seamlessly in mixed designs, despite the constituent regional neural unit differences in core and matrix TC loops.

**Figure 8.**
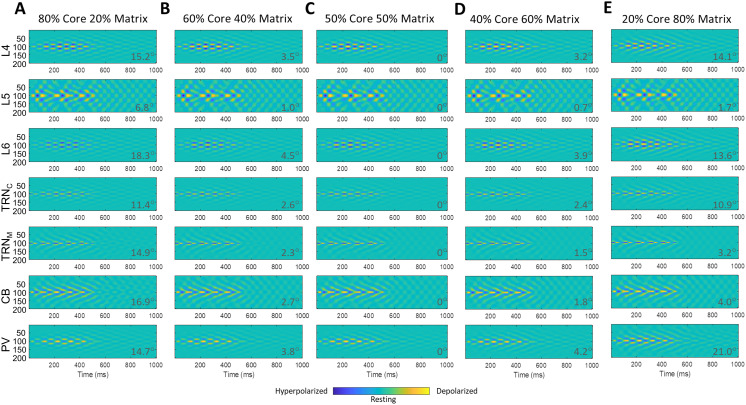
Spatiotemporal dynamics of cortical mixed TC model with different ratios of involved core and matrix. (A–D) The spatiotemporal dynamics of TC neuronal activity with different ratios of mixed core/matrix: 80/20, 60/40, 40/60, and 20/80 are illustrated, with mixing occurring at the level of the cortex. The higher the ratio of core/matrix, the more core-like were the spatiotemporal dynamics, that is, focal in space and less smeared over time. Conversely, the higher the ratio of matrix/core, the more matrix-like were the spatiotemporal dynamics, that is, more diffuse in space and smeared over time. This trend could be seen in all (in particular core) regions (L4, L6, TRN_C_, and PV) involved in the mix TC loop; the spatiotemporal dynamics of matrix specific regions (L5, TRN_M_, and CB), more or less, remained the same, that is, diffuse and smeared across different ratios of mixing, as if matrix kept its diffuse spatiotemporal dynamics due to its wider horizontal (within cortical laminae) connectivity and depending on the ratio of core/matrix in the mix, the activity of core regions could be more or less matrix-like. To further compare the effects of mixing, we set 50–50% mixing level in panel C as the baseline (angle ***θ***: *ArcCos* of cosine similarity = 0°). Comparison of ***θ*** values between panels provides an estimate of the magnitude of blending effects, expressed as rising deviation of the angle ***θ*** from 0°. Higher deviation of the angle ***θ*** from 0° indicated increased dissimilarity from the baseline in panel C.

**Figure 9.**
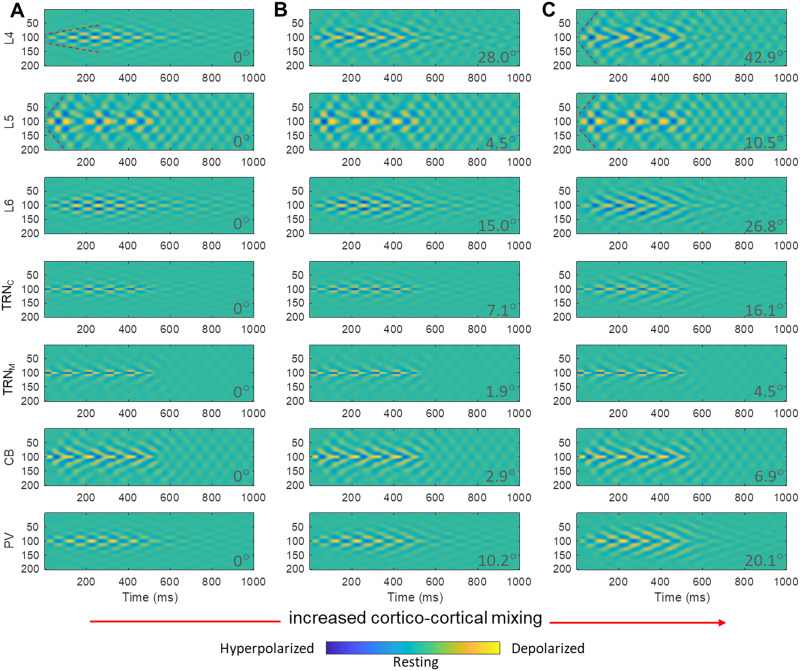
Spatiotemporal dynamics of TC model with different synaptic efficacy of cortical mixing of core and matrix. The spatiotemporal dynamics of the TC model plateaued after reaching increased synaptic efficacy in cortical mixing. The plateau synaptic efficacy being used was 30% (A), 60% (B), and 90% (C). Similar to Figure 8, matrix model neurons kept their spatiotemporal dynamics, and the response of core model neurons resembled the response of matrix model neurons as the efficacy of mixing gradually increased. Model core cortical layer 4 (L4), in 90% mixing level (C), compared to 30% mixing level (A), showed faster activity propagation across neurons indicated by higher slope (i.e., number of neurons per millisecond) of red dotted marks. This shows that mixing core with matrix impacts core signal propagation rate. On the other hand, matrix fast propagation rate remained relatively the same across different mixing levels of core and matrix, indicated by the same slope of red dotted marks in panels A and C L5 panels. To further compare the effects of mixing, we set cortical mixing level in panel A as the baseline (30% of the plateau of the synaptic efficacy), with an estimated angle ***θ*** (*ArcCos* of cosine similarity) = 0°. Comparison of ***θ*** values in panel A with B and C provides an estimate of the cumulative blending effects of gradually increasing mixing levels in B and C, expressed as rising deviation of the angle ***θ*** from 0°. Higher deviation of the angle ***θ*** from 0° indicated incremental increasing dissimilarity of B and C from A.

Figure 8 shows a range of cortical core/matrix mixing ratios from 80% core/20% matrix to 20% core/80% matrix in five steps. The level of core and matrix involvement influenced the spatiotemporal dynamics of spindling tendency in the network. Mixed TC loops were mostly influenced by matrix toward a diffuse spatial and smeared over time spindling tendency (hyperpolarizations and rebound depolarizations), highlighted by the increased deviation from 0° (50–50% mix) of the angle ***θ*** (*ArcCos* of cosine similarity), at each panel bottom right, which indicates increased dissimilarity. Neurons of the model matrix TC loops (e.g., in L5 or CB) influenced the spindling of core neurons (e.g., in L4 or PV) in the mix, while retaining a relatively unaffected diffuse and smeared in time pattern of spindling. The same relationship of increased dissimilarity with increased deviation of the angle ***θ*** from 0° was observed in all mixing scenarios examined (Figure 7, Figure 8, and Figure 9).

Thalamic or cortical mixing produced relatively similar results. The difference was the granularity of the impact of mixing. In cortical mixing, the effect was fine grained (Figures 8 and 9), in which the graded increment of efficacy or ratio of mixing resulted in graded change in the spatiotemporal dynamic of response. On the other hand, in thalamic mixing, the initial increment of efficacy or ratio of mixing brought the spatiotemporal dynamic of mix to the max/plateau level quickly, acting as a coarse tuning knob (Figure 7).

## DISCUSSION

We developed a circuit-based TC computational model, with distinct core and matrix loops, based on detailed neuroanatomical organization and connectivity data of TC circuits in primates that can simulate sleep spindles. We additionally simulated mixed TC loops through novel multilevel cortical, thalamoreticular, and corticoreticular mixing to investigate the functional consequences of different ratios of core and matrix node connectivity contribution to spindle dynamics. Importantly, our rate-based model, for the first time to our knowledge, included and examined the role of local thalamic inhibitory interneurons, and direct layer 5 projections of variable density to TRN (L5-TRN) and thalamus. Simulations of our rate-based model circuit showed the following: (a) increased local inhibition in the thalamus or (b) increased TRN inhibition of core and matrix thalamic neurons could enhance spindle generation and sustain spindle activity for longer periods; (c) the nature of spindles in matrix was more diffuse compared to core, with the mix type showing intermediate properties in agreement with hypotheses that spindles can be classified in core-generated, matrix-generated or mixed types, depending on the neuroanatomy of pathways involved in their generation (Piantoni et al., 2016); (d) the L5-TRN projection enhanced spindle generation and propagation; (e) spindle power could be modulated based on the level of cortical feedback and involvement in model core versus matrix; and (f) matrix TC spindles synchronized their spatial propagation early on, whereas core TC spindles tended to remain spatially focal. In the mix model, the activity of core neurons synchronized and inherited matrix synchronization.

### Connections From Cortical L5 to TRN Are Necessary for Heterogeneity of Spindle Oscillations, Especially for Matrix and Mix Spindle Generation

Projections from L5 to TRN have only recently been shown directly or indirectly for some cortical areas and at variable levels (Hádinger et al., 2022; Prasad et al., 2020; Zikopoulos & Barbas, 2006, 2007a). In primates, several thalamic nuclei, especially those connected with prefrontal cortices, receive anywhere from 20 to 50% of their cortical projections from layer 5 pyramidal neurons (Xiao et al., 2009). These thalamic nuclei, which receive projections from pyramidal neurons in cortical layers 5 and 6, participate in matrix TC loops, in addition to typical core TC circuits (Zikopoulos & Barbas, 2007b). These robust projections from layer 5 terminate as axonal branches with a mix of small and large axon terminals that form synapses with thalamic projection neurons and interact with ionotropic glutamate receptors (Reichova & Sherman, 2004; Zikopoulos & Barbas, 2007b). In primates, these types of axons that contain large or a mix of large and small axon terminals are also found in TRN regions that are connected with prefrontal cortices, account for about 38% of all prefrontal axons, and most likely originate in cortical layer 5. These types of axon terminals are not seen in pathways from sensory cortices to TRN in primates (Zikopoulos & Barbas, 2006).

Our simulations showed that the cortical projection to TRN is a critical circuit component that determines the spatiotemporal dynamics of spindle propagation and underlies the heterogeneity of spindles, observed in functional and physiological studies of the human brain (Bonjean et al., 2011, 2012; Mak-McCully et al., 2014, 2017). The L5-TRN connection, in particular, appears essential for the generation of matrix and consequently mix spindles. With regard to mix spindle dynamics, we examined, for the first time, mixing of the core and matrix connections at multiple levels. Our findings suggest that mixing, both at the thalamoreticular level (CB^+^ and PV^+^ thalamic projection neurons sending their signals to TRNc and TRNm, respectively), or at the corticoreticular level (L5 and L6 pyramidal projection neurons innervating TRNc and TRNm, respectively), can act as a coarse knob for spindle control. That is because the spindle generator (TRN) is targeted in the mix, and, as such, the spindle dynamics change significantly, even after low levels of mixing; hence, we have a narrow dynamical range for mixing. Conversely, cortical mixing can act as a fine knob. This is because, mixing at a level not directly involved in the generation of spindles (i.e., cortex), can be done with a broad dynamical range (fine knob), because the cortex is entrained by the spindle generator level (thalamoreticular), rather than being a generator itself. Cortical mixing is likely the prevalent mode of mixing of TC circuits, because it relies on interlaminar connectivity that constitutes the main component of cortical microcircuitry, that is, projections from neurons in L3/4 to pyramidal neurons in L5, or core cortical nodes in L4 and L6 receiving L1-3 signals that are also influenced by a wide kernel of matrix inputs (Krishnan et al., 2018; Thomson & Lamy, 2007; Yoshimura, Dantzker, & Callaway, 2005). To this effect, recent physiological studies have highlighted the laminar heterogeneity of human sleep spindles (Hagler et al., 2018; Krishnan et al., 2018; Thomson & Lamy, 2007; Yoshimura et al., 2005).

### Local Thalamic Inhibition Increases Ability to Generate, Sustain, and Propagate Spindle Activity

Our simulations showed that increased local inhibition in thalamic nuclei, which can hyperpolarize either core or matrix TC projection neurons, could enhance spindle generation and sustain spindle activity for longer periods. Importantly, GABAergic local circuit neurons are abundant in all the dorsal thalamic nuclei of primates, and may constitute about 40% of all neurons in the human thalamus (Arcelli et al., 1997; Hunt, Pang, & Jones, 1991; Smith, Seguela, & Parent, 1987), but are not present in all the thalamic nuclei of different mammalian species, and are virtually absent in the thalamus of rodents, with the exception of few first-order thalamic nuclei like the lateral geniculate nucleus (Arcelli et al., 1997; Barbaresi, Spreafico, Frassoni, & Rustioni, 1986; Gabbott, Somogyi, Stewart, & Hamori, 1986) and the ventral posterior nuclei (Simko & Markram, 2021). The importance of local thalamic inhibition has not been explored in most experimental and computational studies of TC circuits or sleep spindles, with few exceptions (Arcelli et al., 1997; Joyce et al., 2022; Sherman, 2004; Timbie et al., 2020). There is agreement that local inhibition, which is especially abundant in primates, adds complexity to the synaptology and circuitry of the thalamus, in part by facilitating widespread formation of triadic synaptic glomeruli in most thalamic nuclei (Arcelli et al., 1997; Joyce et al., 2022; Sherman, 2004; Timbie et al., 2020), and likely affects the functional properties of TC circuits that can modulate attention, memory consolidation, and spindle tendency.

Importantly, the ubiquitous presence of GABAergic local circuit neurons in the primate thalamus may provide alternative wiring avenues for the construction of open loop circuitry linking TRN with the thalamus (Figure 1B; also see, e.g., Figure 9 of Barbas & Zikopoulos, 2007, and Figures 5 and 9 of Zikopoulos & Barbas, 2007a). Several studies suggest that the open loop model of TRN-thalamic interactions, in which TRN neurons are excited by a thalamic neuron and, in turn, inhibit a different thalamic neuron or an inhibitory interneuron, can create a tunable filter that may be modified by extra-TRN influences, like corticoreticular pathways (Brown et al., 2020; Willis et al., 2015). On the other hand, closed loop TRN-thalamic connectivity, in which TRN-thalamic neuron pairs are reciprocally connected, allow a short window for initial excitation of a TC neuron followed by inhibition, preventing summation and faithfully transmitting high-frequency signals to the cortex (Steriade & Deschenes, 1984). Since both circuits are found in the primate thalamus, we modeled TRN-thalamic interactions using a novel hybrid loop design (Figure 1C) that can flexibly simulate either open or closed loops. The hybrid loop design we implemented represents the spread of TRN-thalamic connectivity strength using a bilateral Gaussian curve system. Our architecture resembles a closed loop functionally, when the bilateral Gaussian spread of connectivity strength is spatially symmetric, which means that the resulting Gaussian peak of connectivity strength is to the thalamic neuron which directly excites the TRN neuron. In other words, a closed loop in our model is composed of two open loops in opposite directions (balanced). Conversely, when the connectivity spread is either spatially asymmetrical or follows a shifted Gaussian, then our circuit architecture resembles an open loop. The hybrid loop TRN-thalamic connectivity design used in this study can be a powerful tool to further explore open versus closed loop architecture and interactions in the thalamus and other systems in future studies.

### Increased Cortical Influence on TRN Versus Thalamus Affects the Tendency to Generate Spindles in Opposite Ways

Previous studies have shown that TRN neurons are tuned to be more responsive to cortical than thalamic inputs, based on specialized synaptic-receptor interactions (Golshani, Liu, & Jones, 2001; Liu & Jones, 1999). This suggests that changes in cortical input may affect differentially TC versus TRN neurons. Our simulations show that increased ratio of core or matrix TC neuron activation, due to increased cortical feedback to TC that is not accompanied by equivalent increase of cortical feedback to TRN, can reduce a reverberating tendency to promote spindle duration and sustaining. In contrast, increased cortical feedback to TRN neurons can reverse the balance, and increase the spindle tendency (not shown), consistent with findings showing that increasing the corticoreticular conductance (i.e., g(PYR ➔ TRN)) can result in total spindle duration increase (Bonjean et al., 2011), and in line with the spindle generator properties of TRN neurons. However, recent studies have also suggested that depending on the state of cortical and thalamic neuronal activity, high levels of cortical input to TRN may, in some cases, disrupt thalamic spindling (Bonjean et al., 2011, 2012; Mak-McCully et al., 2014, 2017), highlighting the complexity of the TC circuit interactions in primates.

### Conclusions and Implications for Future Studies

Thalamocortical circuits are organized into diffuse matrix and spatially selective core components that are mixed in different ratios throughout the brain (Piantoni et al., 2016). TC circuits can indirectly propagate signals from one cortical area to another, in concert with or in addition to direct corticocortical pathways, forming a framework for rhythmic brain activity, including signal and spindle propagation across cortex (Brown et al., 2020; Halassa & Sherman, 2019; Jones, 2001; Sherman, 2012; Sherman & Guillery, 2013). Moreover, thalamocortical and cortical connectivity influence sleep spindle properties (Krishnan et al., 2018). Therefore, changes in spindle dynamics can be considered a litmus test for typical thalamocortical circuit connectivity and for disruptions underlying neuropathology, because the latter can also impact spindling tendency. For example, medicated individuals with chronic schizophrenia show deficits in spindle density (Ferrarelli et al., 2007; Manoach et al., 2010), which parallel impaired memory consolidation during stage 2 sleep (Goder et al., 2015; Wamsley et al., 2012) and increased thalamocortical functional connectivity (Baran et al., 2019; Manoach & Stickgold, 2019). Moreover, spindle density and duration are significantly decreased in autism spectrum disorders (ASD) and show negative correlation with sleep-dependent memory consolidation (Farmer et al., 2018; Mylonas et al., 2022). Both disorders differentially affect sensory processing, attention, and emotional processing likely involving at variable degrees first-order primary networks that participate in core TC circuits, or high-order association networks that participate in matrix or mix TC circuits. Therefore, changes in spindle dynamics can also point to the imbalance of core and matrix involvement in disorders such as schizophrenia and ASD, pinpointing specific TC loops, circuit nodes, cell types, and underlying mechanisms that are likely disrupted. These disruptions are manifested through distinct symptomatology, for example, distractibility and difficulty focusing attention is a hallmark of schizophrenia (Braff, 1993; Luck & Gold, 2008), whereas difficulty switching attentional focus and sensory overresponsivity are often seen in ASD (Allen & Courchesne, 2001; Cheung & Lau, 2020; Fan et al., 2012; Green & Ben-Sasson, 2010; Keehn, Müller, & Townsend, 2013; Marco, Hinkley, Hill, & Nagarajan, 2011). Future studies can use the modeling framework we developed to further investigate how key circuit organization features in primates, including thalamic inhibition, locally or through TRN, and extensive L5-TRN projections, can change spindle generation and their spatiotemporal propagation in health and in disease.

## ACKNOWLEDGMENTS

We would like to thank Dr. Yohan John and Natalia Matuk for useful discussions and comments.

## SUPPORTING INFORMATION

Supporting information for this article is available at https://doi.org/10.1162/netn_a_00311.

## AUTHOR CONTRIBUTIONS

Arash Yazdanbakhsh: Conceptualization; Data curation; Formal analysis; Funding acquisition; Investigation; Methodology; Project administration; Resources; Software; Supervision; Validation; Visualization; Writing – original draft; Writing – review & editing. Helen Barbas: Conceptualization; Investigation; Writing – review & editing. Basilis Zikopoulos: Conceptualization; Data curation; Formal analysis; Funding acquisition; Investigation; Methodology; Project administration; Resources; Supervision; Validation; Visualization; Writing – original draft; Writing – review & editing.

## FUNDING INFORMATION

Basilis Zikopoulos, National Institute of Mental Health (https://dx.doi.org/10.13039/100000025), Award ID: R01 MH 118500. Arash Yazdanbakhsh, National Institute of Mental Health (https://dx.doi.org/10.13039/100000025), Award ID: R01 MH 118500.

## TECHNICAL TERMS

Sleep spindles: Widespread brain oscillations with a frequency of 7–15 Hz and duration of 0.5–3 sec, associated with the beginning of deep NREM sleep, learning, and memory consolidation.
Thalamic reticular nucleus (TRN): Inhibitory nucleus that surrounds the entire thalamus filtering thalamocortical communications through selective inhibition of thalamic neurons.
Core thalamocortical (TC) circuits: Circuits between parvalbumin-expressing thalamic projection neurons (PV^+^) that innervate focally the middle cortical layers and receive input from cortical pyramidal neurons in layer 6.
Matrix thalamocortical (TC) circuits: Circuits between calbindin-expressing thalamic projection neurons (CB^+^) that send widespread innervation predominantly to the upper cortical layers and receive input from cortical pyramidal neurons in layer 5.
Local thalamic inhibition: Inhibition of thalamic projection neurons by local thalamic interneurons that are present in the primate thalamus.
Mixing of core and matrix networks: The two parallel core and matrix loops can be isolated or cross-connect at multiple nodes either within the thalamus (thalamoreticular and corticoreticular cross-connections) or the cortex (corticocortical cross-connections).
Open loop circuits: In open loop connections, TRN neurons do not directly inhibit the thalamic neurons that excited them. Gaussian connectivity strength from TRN to thalamus resembles bilateral but asymmetric open loops with opposite open directions (not balanced). Due to spatially asymmetric bilateral Gaussian spread of connectivity strength, the circuit is composed of two unbalanced open loops in opposite directions therefore our architecture is functionally similar to an open loop.
Synaptic efficacy: The weight or strength of a synaptic connection quantified as a single number, which can be adjusted to increase or decrease.
Triadic synaptic glomeruli: Specialized synaptic arrangements seen in the thalamus where an extrathalamic excitatory terminal innervates the dendrite of a thalamic projection neuron and the dendrite of a local inhibitory interneuron, which is, in turn, presynaptic to the same thalamic projection neuron dendrite. Note that dendrites of local thalamic inhibitory interneurons contain vesicles and can form inhibitory synapses.
Closed loop TRN-thalamic connectivity: In closed loop connections, TRN neurons directly inhibit the thalamic neurons that excite them. Gaussian connectivity strength from TRN to thalamus resembles bilateral and symmetric open loops with opposite open directions (balanced). Due to spatially symmetric bilateral Gaussian spread of connectivity strength, the circuit is composed of two open loops in opposite directions (balanced) therefore, our architecture is functionally similar to a closed loop.
Hybrid loop TRN-thalamic connectivity: Bilateral and symmetric or asymmetric Gaussian connectivity design was used to simulate TRN-Thalamic connectivity (closed or open loops, respectively).
